# Ex Vivo Comparison of a UV-Polymerizable Methacrylate Adhesive versus an Inverting Pattern as the Second Layer of a Two-Layer Hand-Sewn Jejunal Anastomosis in Horses: A Pilot Study

**DOI:** 10.1155/2021/5545758

**Published:** 2021-04-04

**Authors:** Augustin Lenoir, Bertrand R. M. Perrin, Olivier M. Lepage

**Affiliations:** ^1^Groupe de Recherche en Médecine et Rééducation des Equidés de Sport, Centre for Equine Health, Ecole Nationale Vétérinaire de Lyon, VetAgro Sup, Université de Lyon, Marcy-l'Etoile 69280, France; ^2^Cohesives, Laboratoire de Recherche et Développement, Université de Bourgogne, Dijon 21000, France

## Abstract

Resection and anastomosis of small intestine during colic can lead to adhesions and recurrent colic. Several methods are available to reduce the rate of adhesions in the postoperative period, such as the use of serosal barriers. Surgical glues form a smooth surface, are fast to apply, and could reduce surgery time when performing anastomosis. A recently developed UV-polymerizable methacrylate adhesive (UV-PMA) is designed to anchor into the biological tissues' top surface offering sealant and a smooth cover over the anastomosis site. This adhesive was used ex vivo on fifteen samples of equine jejunum as the second layer of a two-layer anastomosis (1L-UV-PMA group) and compared to a two-layer anastomosis (simple continuous pattern covered with a Cushing pattern; 2L-CT group), in terms of feasibility, bursting strength pressure (BSP), luminal diameter reduction (LDR), and time of construction. Data were analysed using a paired *t*-test or a chi^2^-test (*P* < 0.05). The results showed no statistical difference in BSP, LDR, or any mode of failure between the two anastomosis types. However, the glue anastomosis formed a tunnel-like anastomosis and shredded under pressure, before apparition of leakage, preventing its usage in clinical cases with this methodology. It was concluded that modification of the technique is warranted before testing in clinical cases. A preprint of a former version of the manuscript is available on researchsquare.com, which was not conducted to print and publication after peer reviewing. Since then, the manuscript has been modified to this current version.

## 1. Introduction

Involvement of the small intestine, mainly jejunum, during emergency exploratory laparotomy is reported in about 34% of cases [1]. Resection and anastomosis is the method of choice when a segment of devitalized small bowel is found.

Several methods of resection and anastomosis of the jejunum have been described, including hand-sewn techniques (one or two layers using Lembert, Cushing, or Gambee patterns, and lately the use of barbed sutures), staples, and biofragmentable anastomosis ring [2–6].

Intra-abdominal adhesions can cause recurrent colic after intestinal surgery in horses [7–10]. Accurate diagnosis of adhesions is difficult and necessitates repeated celiotomy, laparoscopy, or post-mortem examinations. Clinical signs associated with adhesions are scarce and not specific; furthermore, they include recurrent colic within 2 months after surgery, and 18–53% of horses require repeated celiotomy and/or euthanasia [7, 9–11]. Preventive strategies are necessary to reduce the formation of adhesions in the postoperative period, and include the use of abdominal and systemic administration, omentectomy, intraoperative and postoperative lavage, abdominal drains, and serosal barriers [7]. These barriers are used during surgery and can be either high molecular weight solutions, such as sodium carboxymethylcellulose, or the use of a bioabsorbable membrane made from hyaluronate-carboxymethylcellulose, as recently described [7].

Surgical glues are rarely used in veterinary medicine and their plastic properties could be used as a serosal barrier which is similar to the use of a bioabsorbable membrane. Five types of surgical glues are used in human surgery: fibrins and bovine collagen and thrombin, cyanoacrylates, polyethylene glycol, and aldehydes. Fibrins and bovine collagen and thrombin sealants are hemostats [12]. They are essentially used in cardiac and vascular surgeries. Their effectiveness and usefulness remain questionable [13]. However, they are widely used because they are occasionally the last tool available to treat in difficult surgical bleedings [14]. Polyethylene glycol and aldehydes are essentially used in aortic suture sealing. Cyanoacrylates are the only surgical glues that can be considered as adhesives. They are only used to suture small skin incisions (<1 cm) and as bandage over skin essentially because their adhesion is low [15–17]. Cyanoacrylate has been described for gastrointestinal anastomoses in rats, pigs, and dogs [18–20]. Their use in horse abdominal surgery has been limited to inguinal ring closure under laparoscopic guidance [21].

Unfortunately, hemostatic, sealing, and adhesive properties of surgical glues are disappointing. This is mainly due to a low adhesion to biological tissues and a hold over time that is shorter than tissue healing time. An efficient surgical adhesive, able to seal strongly, would be a great tool for surgeons and a major clinical breakthrough. New surgical adhesives solutions are currently widely developed and researched [22–27].

An UV-polymerizable methacrylate adhesive (UV-PMA) (Cohesives, 21000, Dijon, France) is designed to anchor into the biological tissues' top surface offering sealant and has adhesive properties up to ten times greater than commercially available soft tissue glues.

The objective of our study was to compare the UV-PMA with a Cushing pattern as the second layer of a two-layer jejunal anastomosis, in terms of feasibility, sealing properties, luminal reduction, and anastomosis time. We assumed that UV-PMA would have the same mechanical properties and be faster to undertake than a Cushing pattern.

## 2. Materials and Methods

Small intestinal segments were collected from 15 client-owned horses euthanized for reasons unrelated to disease of the gastrointestinal system. Horses were free of any signs of colic within 24 hours before euthanasia and owner consent was obtained as a donation for research. Intestinal segments were harvested immediately after euthanasia. Anastomosis, bursting pressure test, and lumen reduction measurements were all performed within the following 4 hours.

### 2.1. Intestinal Specimens

A segment of 1.5 to 2 meters of jejunum was harvested. Three to five centimeters of mesentery were kept on the mesenteric border. The segment was rinsed with tap water to remove any ingesta and then stored in a saline (0.9% NaCl (Osalia, 75009, Paris, France)) solution at room temperature throughout the study except during anastomosis and testing procedures. Three samples of 30 to 40 cm were obtained from each harvested segment to perform a two-layer hand-sewn anastomosis (2L-CT group), a one-layer hand-sewn anastomosis sealed with an UV-PMA layer (1L-UV-PMA group) and a control segment, not subject to anastomosis (Control group).

### 2.2. Anastomosis Techniques

A two-layer anastomosis and a one-layer anastomosis with application of methacrylate glue were undertaken on each sampled horse by the same ECVS Resident. All intestinal samples for the suture and glue groups were transected at mid-distance from each end with a 60° angle from the mesenteric attachments before performing the anastomosis.

#### 2.2.1. 2-Layer Anastomosis (2L-CT Group)

**Figure 1:**
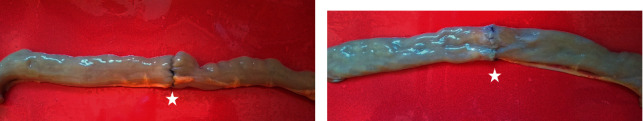
Intestinal samples ready for testing. (a) Double-layer hand-sewn anastomosis. (b) One-layer anastomosis covered by the UV-polymerizable methacrylate adhesive. The star is located at the mesenteric border.

Polyglycolic acid USP 2-0 on a taper cutting needle (Safil, B.Braun Surgical S.A., 08191, Rubí, Barcelona, Spain) was used in a hemicircumferential simple continuous full-thickness pattern. All layers of the intestinal wall were apposed, and the suture was interrupted at the mesenteric and antimesenteric borders to avoid a string-purse effect. Suture bites were taken approximately 5 mm apart and 3 mm from the incised edge. The same suture material was then used in a hemicircumferential Cushing pattern, started at the middle of the first layer sutures (3 and 9 o'clock), in order to avoid knot superposition between the two layers. Bites were placed 3 mm from the first layer and 5 mm apart (Figure 1).

Construction time from the first bite of the second layer to the final knot was recorded.

#### 2.2.2. 1-Layer Anastomosis and UV-PMA (1L-UV-PMA Group)

The same technique as in the 2L-CT group was used for the first layer. After this, the intestinal sample was dried with a gauze swab, before application of the glue.

The adhesive consists of 2 separate layers and is liquid in its initial form. It solidifies with the aid of UV-light at a wavelength of 395 nm.

The first layer of the surgical glue was directly applied on the suture, on a width of about 5 mm on each side of the suture, and was polymerized by the action of the UV LED curing lamp for 30 seconds. Then, the second layer was applied over the first one and polymerized by the action of the UV LED curing lamp for 30 seconds. The intestine was rolled between the surgeon's fingers while the UV lamp was applied to polymerize the glue on the entire circumference of the bowel (Figure 1).

Construction time for glue application was recorded from the end of the first suture to completion of the second 30 sec UV-light period.

### 2.3. Evaluation of Luminal Diameter Reduction (LDR)

Before mechanical testing, each segment was distended to an intraluminal pressure of 20 mmHg, as previously described [5]. Ultrasonography was used to determine luminal diameter reduction for 2L-CT and 1L-UV-PMA groups.

**Figure 2:**
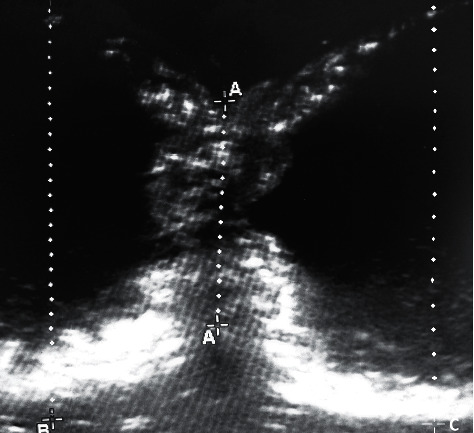
Ultrasound image recorded for luminal diameter reduction. The “*A*” dotted line is at the location of the suture line, the “*B*” dotted line is positioned 2 cm cranial to the anastomosis, and the “*C*” dotted line is positioned 2 cm caudal to the anastomosis.

Ultrasonography was performed using a 6–15 MHz linear transducer (HFL50 with Edge II, FUJIFILM SonoSite, 98021-3904 Bothell, WA, United States). For each group, longitudinal images on the anastomosis were recorded. Care was taken to obtain the largest diameter possible. The samples were held in a linear position by an assistant. Luminal diameter measurements were determined from these images using a linear function of the ultrasound unit, on the anastomosis site (measurement *A*) and at 2 cm proximal and distal to the anastomosis (measurements *B* and *C*, Figure 2).

Mean value of measurements *B* and *C* served as reference for normal luminal diameter. LDR at the anastomosis site was calculated by dividing the luminal diameter calculated at the anastomosis site (measurement *A*) by the mean normal diameter.

(1) $$\begin{array}{c} \text{LDR}=\frac{2xA}{B+C}. \end{array}$$

### 2.4. Bursting Strength Pressure (BSP) Testing

**Figure 3:**
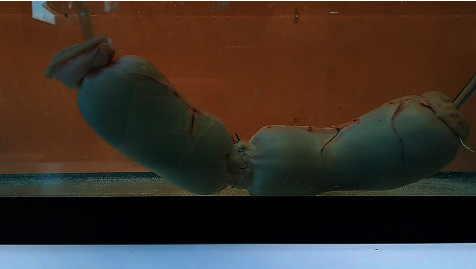
The intestinal segment (1L-UV-PMA group) is submerged in a water tank, ready for bursting strength pressure testing. The infusion set on the left is connected to the roller pump for fluid infusion; the infusion set on the right is connected to the pressure sensor. Note the tunnel-like anastomosis.

After completion of the anastomosis, the intestinal segment was placed in a water tank for bursting strength pressure testing. The same method as previously described was used [3, 5, 6, 28–32]. Briefly, each intestinal segment was submerged in 30 L saline solution within a water tank at room temperature. Infusion sets were inserted at each end of the intestine and a knot was made with a polypropylene string around the intestine to provide a watertight seal between the intestinal wall and the infusion set (Figure 3). One infusion set was connected to a roller pump (BSM-21, Hospal-Baxter, 69330 Meyzieu, France) for fluid delivery. The other infusion set was connected to a T-connector attached to a pressure transducer (MP100A-CE, BIOPAC Systems, 93117 Goleta, CA, United States). The pressure transducer was then connected to a computer to assess real-time pressure within the bowel on a dedicated software (AcqKnowledge, BIOPAC Systems, 93117 Goleta, CA, United States). A balanced electrolyte solution (Hartmann's solution) tainted with methylene blue (2 mL/L, 0.2%) was infused in the bowel at constant rate (700 mL/min, pump's maximum) until failure occurred. Bursting strength pressure (BSP) was determined as the maximal pressure obtained before failure. Failure was first detected as apparition of blue tainted flow inside the saline bath. With time, visual rupture of the intestine could be observed. Every trial was video-recorded and reassessed to describe the mode of failure.

**Figure 4:**
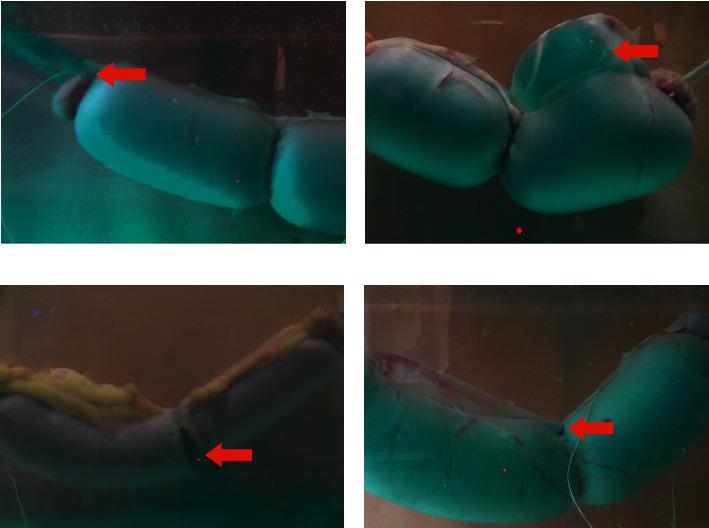
Modes of failure after bursting strength pressure testing. The red arrows show the location of the failure. (a) “Extremity” mode of failure, 2L-CT group. (b) “Mesenteric mucosa” mode of failure, 2L-CT group. (c) “Nonmesenteric suture” mode of failure, 1L-UV-PMA group. (d) “Mesenteric suture” mode of failure, 1L-UV-PMA group.

Modes of failure were recorded as following: “extremities” when the knots around the infusion sets ruptured before the intestine; “mesenteric mucosa” when the mucosa and muscularis ruptured and the intestine ballooned without rupture of the serosa on the mesenteric border of the intestine at a location more than 2 cm away from the suture; “mesenteric suture” when the suture line ruptured at the mesenteric border of the intestine; “non-mesenteric suture” when the suture ruptured at a location different from the mesenteric border (Figure 4).

The same mechanical testing was performed for the control group.

Macroscopic evaluation of the anastomosed segments was performed before and after testing. The anastomosis before and after BSP testing was observed for abnormalities and shredding of the glue, and the mode of failure was confirmed after BSP test by visual assessment.

### 2.5. Statistical Analysis

Descriptive statistics were reported as mean (95% CI). Data was tested for normality with Shapiro–Wilk test.

A paired *t*-test was realized to evaluate a statistical difference in construction time, BSP and LDR.

Mode of failure was assessed using a chi^2^-test between suture-related failures (mesenteric and non-mesenteric suture types of failure) and non-suture-related failures (mesenteric mucosa types of failure).

Statistical analysis was performed using excel software (Excel Office 365 for Windows, Microsoft Corporation, Redmond, WA 98052-6399, United States) and for all statistical tests a *P* < 0.05 was considered significant.

## 3. Results

Horses (6 males, 9 females) had a median age of 11 years (range, 2–27 years). Breeds were represented as follow: 8 Thoroughbred Cross (Selle Français), 3 Thoroughbred, 2 Standardbred, 1 Shetland Pony, and 1 Spanish Horse. LDR was available for 14 horses only due to a technical issue with the ultrasound in the first horse tested. All data were determined to be normally distributed.

### 3.1. Construction Time

Mean (95% CI) 1L-UV-PMA construction times (3.02 min [2.50; 3.55]) were significantly lower compared to 2L-CT (8.09 min [7.59; 8.61]; *P* < 0.001).

### 3.2. Bursting Strength Pressure

Both anastomoses groups had a lower BSP compared to the control group (control: 189.93 mmHg [162.52; 217.34]; 2L-CT: 175.33 mmHg [156.83; 193.83]; 1L-UV-PMA: 170.47 mmHg [146.29; 194.65]), with only the difference between the 1L-UV-PMA group and the control group being statistically significant (*P*=0.04).

### 3.3. Luminal Diameter Reduction

No significant difference was found in LDR between suture (48% [43; 53]) and glue groups (51% [47; 55], *P*=0.26, Table 1).

### 3.4. Mode of Failure

No significant difference in mode of failure was observed in our study (*P*=0.36). However, six (mesenteric suture: *n* = 3; nonmesenteric suture: *n* = 3) and eight (mesenteric suture: *n* = 5; nonmesenteric suture: *n* = 3) segments in the suture and glue group, respectively, ruptured on the suture line (Table 2). All anastomoses showed serosal tearing at the point of at least one suture penetration.

### 3.5. Macroscopic Evaluation

**Figure 5:**
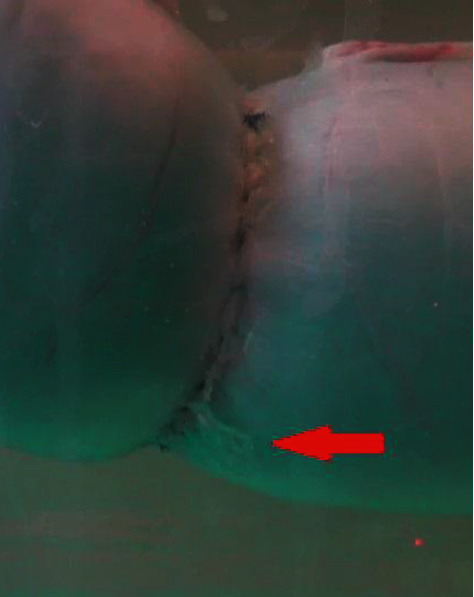
Shreds of glue on the anastomosis line. The red arrow shows a shred of glue erupting from the suture line.

On macroscopic evaluation, the 1L-UV-PMA anastomosis formed a wider anastomosis site than the 2L-CT anastomosis, which looked like a small tunnel instead of a constriction ring. After testing, some of the samples from the 1L-UV-PMA group showed shreds of glue detached from the serosa (Figure 5), while all samples from the 2L-CT group showed tearing of the serosa on the second suture line.

## 4. Discussion

This study evaluated the feasibility of the use of a UV-PMA as the second layer of a jejunal anastomosis. UV-PMA was as effective as ligatures in terms of strength and luminal reduction but quicker to apply. However, macroscopic evaluation revealed the formation of an anastomotic tunnel and shredding of the glue with distension precluding its use for clinical cases so far.

Instead of over-sewing the first hand-sewn layer, we decided to place an UV-PMA. In future research, comparing this method with a single-layer anastomosis would be valuable, as previous reports have shown that a one-layer anastomosis is sufficient to achieve good anastomosis in horses and is faster to perform [5, 28–32]. In our study, a one-layer anastomosis with glue application resulted in a tight-sealed anastomosis, which ruptured at equivalent BSP to a two-layer anastomosis. This first layer hand-sewn anastomosis was mandatory to obtain a good apposition of the different segments before applying the glue.

Values obtained in our study for BSP were close to what has been reported in other studies [3, 5, 29–32]. Some of these showed a significant difference between anastomosed and healthy intestine [3, 5] but not all of them had a control group. Although the BSP difference between the 1L-UV-PMA and the control group was significant, BSP values in our study were far above what occurred clinically in horses in a previous study [33]. Because of its liquid and plastic properties, the UV-PMA glue represents a smoother seal than a suture and could reduce the incidence of adhesions.

LDR showed no statistical differences in our study. Values obtained for both anastomosed groups are higher than the maximal 44.6% described in the literature [1]. The reason for this high luminal reduction might come from suturing the first layer with a full-thickness continuous pattern instead of a submucosal continuous pattern as is more commonly performed [1]. Furthermore, because the intestine was used post-mortem and emptied before suturing, we did not use an intestinal clamp to flatten it before suturing. Intestines were shrunk to minimal diameter when sutured, which could explain the high LDR in our study. This would present a major limitation in the interpretation of the diameter reduction results.

Almost half of the anastomoses ruptured at the suture line, highlighting the importance of careful surgical technique when performing bowel resection and anastomosis in clinical cases. Nieto et al. [31] reported that incorporating the submucosa in the suture line is the most important step when suturing and that incomplete incorporation of this layer would cause failures close to the anastomosis. In our study, there were no ruptures adjacent to the suture line, but failure at the suture line could be caused by the incomplete incorporation of the submucosa as well. The ruptures in our study occurred most of the time at the mesenteric border, as previously reported [5, 28, 30, 32]. This site of weakness may be induced by improper knot placement or inaccurate seromuscular suture placement.

On a visual assessment, the 1L-UV-PMA anastomosis caused a larger constriction on the anastomosis site and was less prone to distension than the 2L-CT anastomosis. Large application (5 mm per side) is mandatory for the glue to anchor into the tissue, which prevents its application only on the suture. In the immediate postoperative period, a constriction of the anastomosis site may interfere with the passage of ingesta. Intussusception has been shown to appear secondary to inflammation or during the postoperative period in humans and horses [34, 35], and the formation of a constriction ring could create a starting point for an intussusception. Using the glue at critical locations, such as at the attachment to the mesentery or at the suture knots, could significantly improve the technique: this could limit serosal inflammation on those points as well as decrease the risk of a constriction ring.

The glue was shredded when the intestines were submitted to high pressures. This response makes the glue unfit for clinical use as it is, and necessitates further investigation of and modification to the application technique. The formation of glue shreds would lead to local reactions and therefore would increase the risk of adhesions, instead of reducing it.

Intestine anastomoses have been reported to be weakest at 3–7 days after surgery [36, 37]. Since we opted for an ex vivo study, we could only evaluate the anastomosis' strength at the time of construction. However, we could not evaluate the anastomosis site's long-term healing, such as inflammation, or other side effects of the glue (e.g., adhesions) within the abdominal cavity.

At this stage, several changes to the glue are necessary to make it suitable for clinical cases. The first glue component was very fluid and tended to spread over the bowel and the surgical area. Extra care should be taken not to spill any glue within the abdomen during surgery. This can be done by adding supplementary surgical drapes to isolate the anastomosis site before application. The second glue component had a more viscous consistency and was easier to apply on the suture line. When submitted to high pressures, the glue kept its initial form and formed a constriction ring, resisting tissue distention at the anastomosis site. Therefore, the tubular shape of the bowel should be considered when polymerizing the glue with ultraviolet light. Under pressure, the glue broke and shredded on the surface of the intestines. The UV-PMA is nonresorbable in its current form, and with the formation of a constriction ring and shreds, the use in live animals is therefore unfit. A permanent constriction site on a regenerating intestine could lead to permanent stenosis and recurrent colic signs. Being able to manufacture a bioresorbable intestinal glue would be a great improvement and would lead to clinical use in all types of horses, with minimal risk of permanent stenosis after anastomosis.

## 5. Conclusions

This study shows that a Cushing pattern or the use of UV-PMA as the second layer of a two-layer jejunal anastomosis has a similar BSP and LDR, and the UV-PMA application is faster to perform than a hand-sewn second layer.

However, the glue layer forms a stiffer and larger anastomosis ring than a hand-sewn anastomosis. In its current form, it has issues regarding its physical properties. Modifications to the application technique need to be evaluated before considering the possibility of making clinical trials.

## Figures and Tables

**Table 1:** Mean values and confidence intervals obtained from the control, 2L-CT, and 1L-UV-PMA groups.

	Control [95% CI]	2L-CT [95% CI]	1L-UV-PMA [95% CI]
BSP (mmHg)	189.93 [162.52; 217.34]^*∗*^	175.33 [156.83; 193.83]	170.47 [146.29; 194.65]^*∗*^
LDR (%)	N/A	48 [43; 53]	51 [47; 55]
Construction time (min)	N/A	8.09 [7.59; 8.61]^*∗*^	3.02 [2.50; 3.55]^*∗*^

^*∗*^Significant difference (*P* < 0.05). 2L-CT: double-layer anastomosis; 1L-UV-PMA: 1-layer anastomosis and UV-polymerizable methacrylate adhesive application; BSP: bursting strength pressure; LDR: luminal diameter reduction; N/A: non-applicable.

**Table 2:** Rupture pattern from control, 2L-CT, and 1L-UV-PMA groups.

	Control	2L-CT	1L-UV-PMA
Extremities	5	3	2
Mesenteric mucosa	10	6	5
Mesenteric suture	N/A	3	5
Antimesenteric suture	N/A	3	3

Nonsuture site	**10**	**6**	**5**
Suture site	**N/A**	**6**	**8**

2L-CT: 2-layer anastomosis; 1L-UV-PMA: 1-layer anastomosis and UV-polymerizable methacrylate adhesive application; N/A: non-applicable.

## Data Availability

The datasets used and/or analysed during the current study are available from the corresponding author on reasonable request.
